# Treatment of Superficial Cutaneous Vascular Lesions: Experience with the Long-Pulsed 1064 nm Nd:YAG Laser

**DOI:** 10.1100/2012/197139

**Published:** 2012-09-17

**Authors:** Kemal Ozyurt, Emine Colgecen, Halit Baykan, Perihan Ozturk, Mehmet Ozkose

**Affiliations:** ^1^Department of Dermatology, Medicine Faculty, Sutcu Imam University, 46100 Kahramanmaras, Turkey; ^2^Department of Dermatology, Medicine Faculty, Bozok University, 66200 Yozgat, Turkey; ^3^Department of Plastic Surgery, Medicine Faculty, Sutcu Imam University, 46100 Kahramanmaras, Turkey

## Abstract

Recent published studies evaluating the long-pulsed 1064 nm Nd:YAG laser for superficial cutaneous vascular lesions have limited subjects and optimal treatment parameters have not been established. To determine the efficacy and safety of the long-pulsed 1064 nm Nd:YAG laser on superficial cutaneus vascular lesions and analyse retrospectively our experience of a 3-year period are the aims of this study. Over the 3-year period, 255 patients were treated [189 female and 66 male; median age 35 (range 7–65) years; Fitzpatrick skin types II-V]. Twenty-six patients with spider angioma, 130 with facial telangiectasia, and 99 with leg telangiectasia were treated. A long-pulsed 1064 nm Nd:YAG laser was used. A test dose was performed at the initial consultation and thereafter patients were reviewed and treated at 4-week intervals for 5 months. Of those patients who completed treatment and followup, 26/26 (100%) of spider angiomas, 125/130 (97%) of facial telangiectasia, and 80/99 (80,8%) of leg telangiectasia markedly improved or cleared. We suggest that the long pulsed Nd:YAG laser is a safe and effective treatment for common superficial cutaneous vascular lesions. However, it is not the first choise to use to treat superficial vessels on the face where depth is not the concern.

## 1. Introduction

The laser treatment of spider angioma, facial telangiectasia, and spider veins, and varicose leg veins comprise a large majority of the patients seen in cosmetic dermatologic surgery practice [1–4]. The most commonly used laser devices for these treatments include the 532 nm potassium titanyl phosphate (KTP), 595 nm pulsed dye laser (PDL), the 755 nm alexandrite laser, intense pulsed light (IPL), and the 1064-nm neodymium yttrium-aluminum-garnet laser (Nd:YAG) [3–7]. Their mechanism of action is based on the theory of selective photothermolysis [8]. For effective laser treatment with selective photothermolysis, the laser needs to penetrate to the depth of the target vessel. In addition, the laser exposure needs to be long enough to cause sufficient slow coagulation of the vessel. The preferential absorption of photon energy by the target chromophore at specific wavelengths of light creates thermal energy, allowing for selective destruction of oxygenated and deoxygenated hemoglobin with only minimal damage to the surrounding tissues. This allows for selective destruction of superficial vascular lesions with minimal scarring [2, 9].

Predisposing risk factors of spider angiomas and facial telangiectasia include skin phototypes I through III, a history of significant sun exposure, and longstanding rosacea. The distribution of spider angiomas is usually focal, with single lesions on the face, neck, chest, and other sun-exposed areas; multiple lesions are present in some systemic diseases. A central feeder arteriole with superficial branches lead to a “spider-like” appearance of these commonly acquired lesions. The arteriole is an aberrant branch of the superficial vascular plexus, directly communicating with dilated superficial capillary branches. The vessel diameter is around 0.1–0.5 mm [1–4, 10].

Leg vein anomalies arise from gravitational dilatation, reflux, and incompetent venous valves. They include spider veins, reticular veins, perforators, tributaries, and varicose veins arising within the system of the greater and smaller saphenous vein. Laser treatment of leg veins is difficult because of a wide range in size and depth, a wide variety in flow, and the many different types of leg vein ectasias. An appropriate work-up should be performed before considering treatment options [4, 11–13]. Currently one of the more common treatment options has been sclerotherapy; it is the injection of sclerosing agents into the vessels. Even though laser technology has gained popularity for treatment of leg telangiectasia, still, sclerotherapy continues to offer superior clinical effect in the majority of cases. Sclerotherapy provides an earlier clinical response and is more cost effective, whereas laser treatment can be used in patients with telangiectatic matting, needle phobia, or sclerosant allergy [11–15]. 

It is crucial to remember the possible systemic associations, even patients may present for cosmetic evaluation of any number of cutaneous vascular lesions. In some cases, prompt diagnosis and treatment may reduce the morbidity and mortality of associated systemic diseases [16, 17]. 

We report herein the long-term clinical results of long-pulsed 1064 nm Nd:YAG laser treatment on three different types of superficial vascular lesions.

## 2. Materials and Methods

Informed consent was obtained from each patient for laser treatment and also permitting to use clinical data for sciential investigations. The present study was conducted by the approval of the local ethics committee.

A high peak power, long-pulse Nd:YAG laser system (GentleYAG, Candela) was used. The laser has a wavelength of 1064 nm, a maximum peak power of 26,33 W, and pulse duration ranging from 0.25 to 300 ms. Maximum fluence that can be delivered by this system is 600 J/cm^2^. Spot sizes are adjustable from 1, 5 to 18 mm at the level of the handpiece. Epidermal cooling is achieved with cryogen cooling system that is administered to tissue by the handpiece. 

Treatment procedures that had been performed are summarized in Table 1. A test dose was performed at the initial consultation, and thereafter patients were reviewed and treated at 4-week intervals. Also starting fluences were summarized in Table 1. However, in some cases, until achieving our desired clinical results, we increased fluence after decreasing the pulse width. In <0, 5 mm leg telangiectasia, maximum fluence was increased up to 500 J/cm^2^ with 1,5 mm spot size. And with the larger telangiectasia, maximum fluence sometimes reached the values of 260–320 J/cm^2^ with 3 mm spot size, according to the vessel size. We used only 1,5 mm spot size in facial telangiectasia, maximum fluence was increased up to 400 J/cm^2^ when necessary. During the treatment, cooling after spray was not used in Fitzpatrick skin types IV and V. Lidocaine plus prilocaine cream was used for topical anesthesia prior to laser therapy in only some patients, with leg telangiectasia, who did not tolerate pain.

Over the 3-year period, 255 patients were treated [189 female and 66 male; median age 35 (range 7–65) years; Fitzpatrick skin types II–V]. Numbers of patients who completed treatment and followup with spider angioma 26 (with diameters up to 1,5 mm), facial telangiectasia (with diameters up to 1,5 mm) 130, and leg telangictasia (with diameters up to 3 mm) 99 were treated. Excluding criteria were diabetes mellitus, cardiovascular diseases, and patients using anticoagulant drugs. Of those patients with spider angiomas 20/26 (77%) were on face, 4/26 (15,3%) on neck, and 2/26(7,7%) on hands.

Within a 5-month period, one month after the first, third, and the 5th laser sessions, grade of response was globally evaluated and categorized on five classifications by the operator independently based on photographic records: clear Category(C)-I, marked improvement C-II, partial response C-III, poor response C-IV, and no change or worsening. Treatment and follow-up data were collated on a hospital automation system (MedData, Integrated Software). All lesions were recorded individually on patient. Numerous and monomorphic lesions were not recorded separately and outcome was reported against a single patient.

SPSS version 17 (SPSS Inc., Chicago, IL, USA) was used for stastical analyses. Standard descriptive statistics were used. For categorical variables, counts and percentages were reported. Chi-square test was used for comparing outcomes of treatments. One-way ANOVA test was used for comparing means of ages of patients with each diagnosis. In all cases, the statistical significance level was set at *P* < 0.05.

## 3. Results

Almost all patients tolerated the procedure moderately well, although pain was a limiting instances in treating leg telangiectasia. It was noted that involution progressed more slowly in vessels of larger diameter and especially on the legs. We generally observed an urticarial reaction associated in the treated site, immediately after treatment. Swelling was self-limited and resolution expedited with the application of a mid potency topical corticosteroid (mometasone furoate ointment). In a few patients with rosacea and one with chronic urticaria, severe urticarial reactions were observed on all over the face, in 24 hours after treatment of facial telangiectasia. Those patients were administered wet dressings with a potent topical corticosteroid for a few days. Portions of larger vascular lesions on legs sometimes darkened and hardened from focal thrombosis, evident some days, weeks, and rarely up to 5 months after treatment. This effectively resolved with time. Rarely, transient side effects were observed, namely, postinflammatory hyperpigmentation, bulla formation, superficial erosions, and crusting. Side effects were summarized in Table 2. 

No statistical difference was observed among the ages of patients with spider angioma, facial, and leg telangiectasia (*P* > 0,05). As a result of the first session; C-I observed in 10/26 (38,4%) of spider angioma, 8/130 (6,15%) in facial telangiectasia, and 4/99 (4.04%) in leg telangiectasia. The differences of improvements was statistically significant (*P* < 0,05). According to results of the third session, C-I in spider angioma were 26/26 (100%) and 57/130 (43,8%) and 21/9 (21,21%) in facial telangiectasia and leg telangiectasia, respectively. Finally, at the end of the 5th session, category-I was observed; 125/130 (97%) and 80/99 (80,8%) in facial telangiectasia and leg telangiectasia, respectively (Table 3).

**Figure 1:**
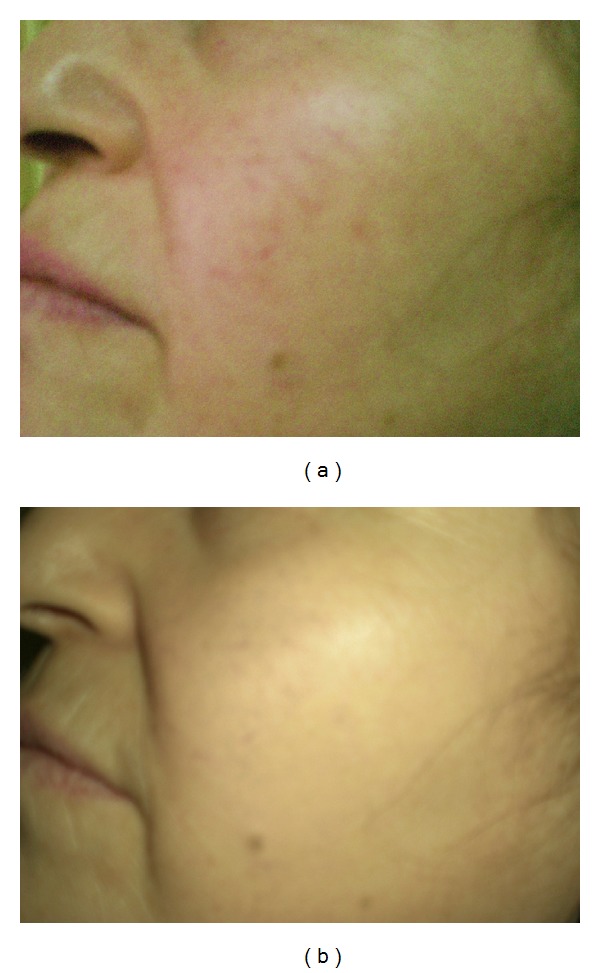
(a) Facial telangiectasia of a patient before treatment, (b) facial telangiectasia in the patient after fifth session of the treatment, Category-I (cleared lesion).

**Figure 2:**
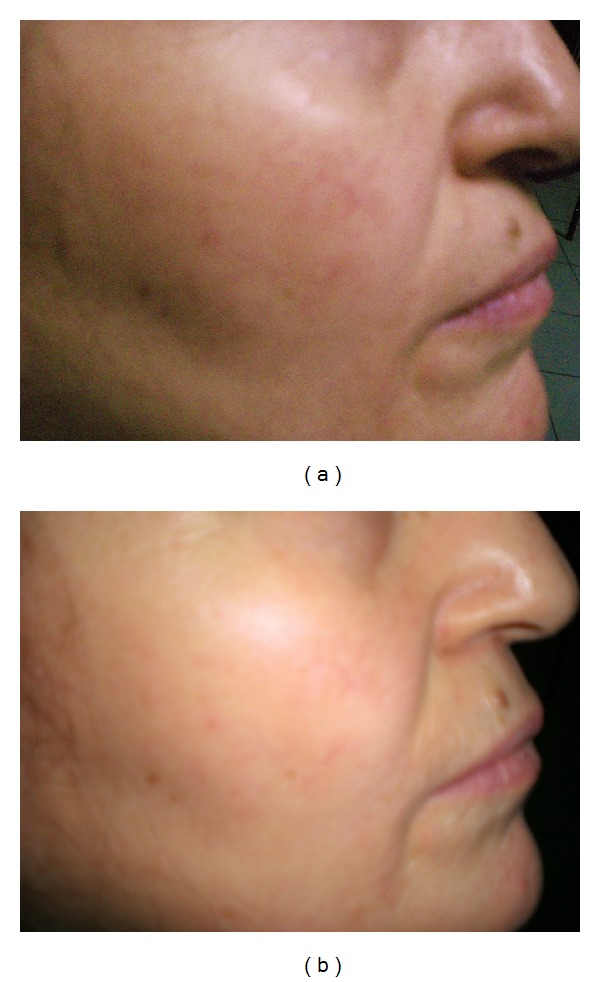
(a) Facial telangiectasia of a patient before treatment, (b) facial telangiectasia in the patient after fifth session of the treatment, Category-I (cleared lesion).

**Figure 3:**
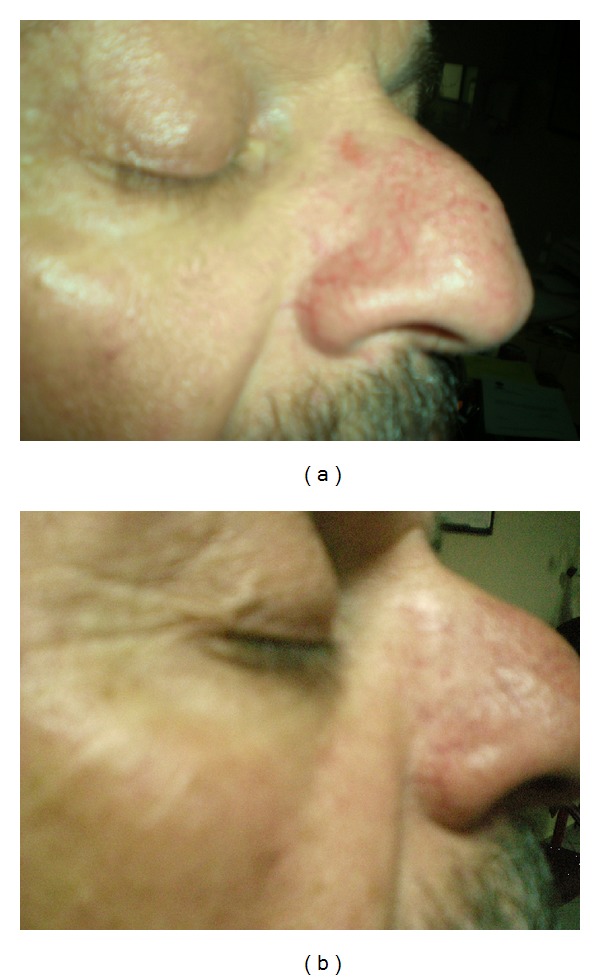
(a) Facial telangiectasia of a patient before treatment, (b) facial telangiectasia in the patient after third session of the treatment, Category-II (marked improvement).

**Figure 4:**
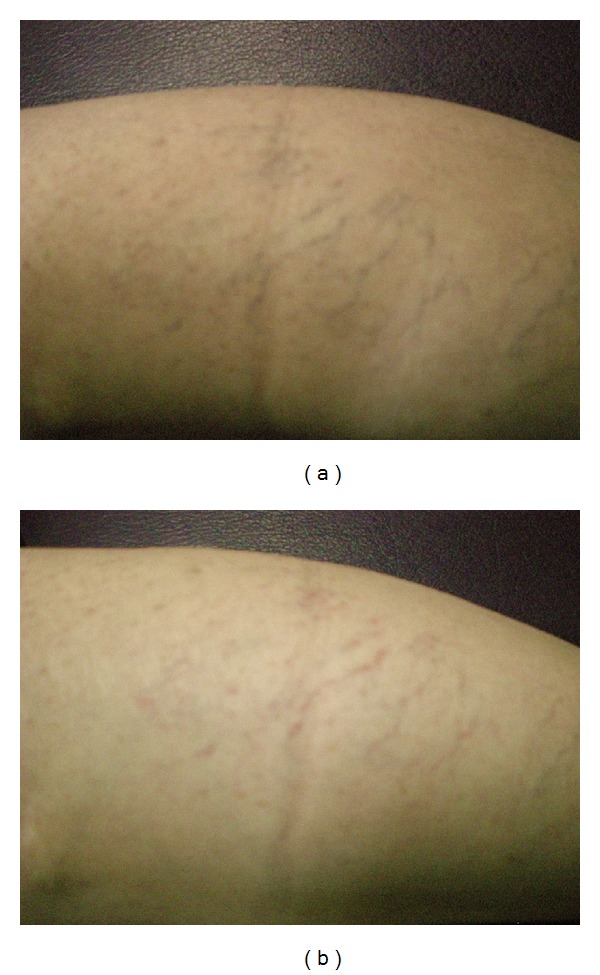
(a) leg telangiectasia of a patient before treatment, (b) leg telangiectasia in the patient after fifth session of the treatment, Category-III (partial response).

 Examples of C-I, C-II, and C-II improvements are illustrated in Figures 1, 2, 3 and 4. Of special interest 100% (26/26) patients of spider angioma improved at the end of the third session. However, neck location 4/26 (15,3%) and hand location 2/26(7,7%) of spider angioma were generally more recalcitrant to the treatment. These 6 patients represented as C-III, in the results of first session. And another interesting observation was noticed in results of first session that; there were 40/130 (30,7%) category-II in facial telangiectasia and 10/99 (10,1%) in leg telangiectasia. In the patients with facial telangiectasia, marked improvements were more than leg telangiectasia (*p* < 0,05). After the 5-month period, some patients had clinical responses of category-II and C-III and subsequently had two or more treatment sessions. These results are not demonstrated in Table 3.

## 4. Discussion

A 2004 publication [18] noted that, comparative studies of laser efficacy/safety were urgently required for superficial cutaneous vascular lesions, in that laser treatment had been carried out in the absence of significant published research. In spite of, some comparative studies cited in the literature [15, 19, 20] we think that the problem is still going on. Beside of this, there are numerous variables for evaluating laser in treatment of superficial cutaneous vascular lesions. When choosing a laser for treating vascular lesions, the following determinants need to be considered: vessel depth and diameter, laser wavelength, pulse width, and, to a limited extent, spot size. The wavelength used needs to have sufficient penetration depth for the target vasculature. The longer wavelength yields deeper penetration yet also requires higher fluences for efficacy. The pulse duration for laser treatment of vascular lesions depends on the target vessel diameter [9, 21].

In our study for treatment procedure of leg telangiectasia between 1–3 mm diameters, we began 60 ms pulse duration with fluence of 200 J/cm^2^ (Table 1). We increased the fluence gradually when outcomes were undesirable. Our experience is compatible with the study of Clark et. al [10] in which KTP frequency doubled Nd:YAG was used for leg telangiectasia. As mentioned in the study [21], in practical applications, we experience some discordance with previous model of selective photothermolysis for in clinical practice pulse durations of <100 ms are used. Secondly, while clinical results are considered satisfactory in most cases, there is no agreement on an optimal pulse duration, fluence, or spot size for the particular Nd:YAG laser used to treat the lesions. A review of the literature indicates that a wide range of parameters is applied clinically. In particular, fluences range from 90 to 400 J/cm^2^, pulse durations may vary from 10 to 100 ms and spot sizes from 1 to 10 mm in order to coagulate leg veins of 0,1–4 mm.

Cases or some clinical series reported that PDL is the initial method of choice [5, 6, 22]. Various centres are also using the Nd:YAG laser for cutaneous vascular lesions using the high wavelength penetrance to also control deeper vessels [7, 23, 24]. Clark et al. [10] reported the use of the Nd:YAG laser in the treatment of superficial cutaneous vascular lesions. They treated 246 lesions in 204 patients, the most commonly treated lesions being spider angioma (102) and telangiectasias (102). They reported marked improvement or clearance in 84% of lesions. These are obviously very good results in a large series of patients and compatible with our series. Goldberg and Meine [20] in 1999 reported on 40 patients with facial telangectasia. They gave a single treatment with four different Nd:YAG lasers and reported, equal and good to excellent improvements in all lasers. Eremia and Li [25] treated face veins with the Nd:YAG laser in 17 patients and reported a greater than 50% improvement in all patients. Thus, our results of first session treatment of facial lesions are compatible with other single-treatment facial series. In the study of Major et al. [26], desirable improvements were observed with a single session in facial telangiectasia with Nd:YAG laser. But three to five sessions were needed for leg telangiectasia. Another study also concluded that Nd:YAG is a good method for treatment of facial telangiectasia [27]. 

Recent studies showed that the role of lasers and light sources in treating lower extremity blood vessels has not been as successful as in treatment of facial telangiectasia to date [26–30]. These results are compatible with our outcomes of study. There are several reasons for this disparity. Increased hydrostatic pressure on the lower extremities may lead to less effective photothermal destructive coagulation. Anatomic considerations are also important, in that lower extremity blood vessels are in a deeper location, have thick surrounding adventitial tissue, and increased basal lamina as compared to facial telangiectasia. 

Indeed a Nd:YAG 1064 nm laser is not the first choice to treat superficial vessels on the face, where depth is not the concern. Also, treating this wavelength for superficial vessels of small diameter with such high fluences have risk for injury. The ability of laser device to adjust fluence and pulse duration independently may decrease risk. In this study, we did not observe significant side effects in facial lesions. However, we do not suggest an Nd:YAG 1064 nm laser as the first choice to treat facial superficial cutaneous lesions. 

R. A. Weiss and M. A. Weiss [31] gave a single treatment to 30 patients for leg veins and achieved 50–75% improvement. Omura et al. [28] used a single treatment with the Nd:YAG laser in 20 patients for reticular veins and showed a 76–100% improvement in 67% patients. They performed treatment of 1–3 mm diameters of vessels at fluences of 100 J/cm^2^ and 50 ms pulse duration. These results are different from our results. We began 60–180 ms with fluence of 180 J/cm^2^. In our series at the result of the first session, cleared lesions C-I of leg telangiectasia was only 4/99 (4,04%), C-II 10/99 (10,1%), and C-III 40/99 (40,4%). Difference may be related to limited subjects of those series.

 In a study, it has been reported that good to excellent results were observed after Nd : YAG than after IPL. Patients with telangiectasia, cherry angiomas, or leg veins <1 mm were more satisfied after IPL, while those with leg veins >1 mm were more satisfied after Nd:YAG [15] Another study declared that Nd:YAG was superior to both diode laser and alexandrite in treating leg telangiectasia. Also they concluded that there were more problems with alexandrite laser than the others [19]. In contrast to this conclusion in series of Kauvar and Lou [32], alexandrite laser was maintained as producing excellent clearance of telangiectasia and reticular veins of the leg with minimal adverse effects. Loo and Lanigan [33], in a review article, mentioned the use of both the ND:YAG and the pulse dye laser in the treatment of telangiectatic leg veins. They reported similar clearance rates of between 50 and 75% for both modalities of lasers; however, lower fluences were used for the PDL (24 J/cm^2^), than for the Nd:YAG laser. 

In our study, pain disturbed patient compliance to the treatment, this experience was minimal in facial telangiectasia and spider angioma, and compatible with two studies [10, 20]. But differently Anwar and Sharpe [27] found pain to be more of an issue on the face. They discussed that the cause of low pain in the study of Clark et al. [10] should have been related to lower fluence. In our idea pain is related with anatomic location depth, and diameter of lesions. Many researchers have reported noticeable pain, which extends from mild to severe. In light of the wide variance in clinical laser, there continues to be ongoing discussion in the field of laser science as to which laser parameters are optimal to treat leg veins effectively, while maintaining a low side-effect profile.

## 5. Limitations

Being a retrospective analysis study is the most important limitations of this study. And we did not compare the efficacy according to the diameters of vessels. 

## 6. Conclusion

Our experience has demonstrated that the Nd:YAG laser is an effective treatment for most common superficial vascular lesions. However, it is not the first choice to use to treat superficial vessels on the face where depth is not the concern. The ability to adjust fluence and pulse duration independently was a particularly useful feature of the GentleYAG, Candela.

## Figures and Tables

**Table 1:** Treatment procedures according to type and diameter of lesions.

Type of lesion/diameter (mm)	Spot size (mm)	Pulse duration (ms)	Starting fluences J/cm^2^	Cooling DCD spray/delay (ms)
Spider angioma	1.5	20	340	10 ms spray/30 ms delay 10 ms post spray
Facial telangiectasia <0.5 mm	1.5	20	340	10 ms spray/30 ms delay 10 ms post spray
Facial telangiectasia 0.5–1.0 mm	1.5	40	250	10 ms spray/30 ms delay 10 ms post spray
Leg telangiectasia <0.5 mm	1.5	20–40	360	10 ms spray/20 ms delay 10 ms post spray
Leg telangiectasia <1.0 mm	3	40–60	220	15–20 ms spray/20 ms delay 10 ms post spray
Leg telangiectasia <1.5 mm	3	40–60	200	15 ms spray/20 ms delay 10 ms post spray
Leg telangiectasia 1.5–3 mm	3	180	180	15 ms spray/20 ms delay 10 ms post spray

**Table 2:** Number of patients with side effects according to type of lesions.

Type of side effects	Spider angioma *n* = 26	Facial telangiectasia *n* = 130	Leg telangiectasia *n* = 99
Pain and erythema	5 (19.2 %)	30 (23 %)	80 (80.8 %)
Severe urticarial reaction	—	3 ( 2.3 %)	—
Focal thrombosis	—	—	7 (7.1 %)
Bulla formation	—	—	2 (2.1 %)
Erosion and crusting	2 (7.6 %)	3 (2.3)	6 (6.1 %)
Transient postinflammatory hyperpigmentation	1 (3.8 %)	3 (2.3)	14 (14.1 %)

**Table 3:** Efficacy results of patients after laser session according to type of lesion.

	Results of first session				Results of third session				Results of 5th session			
Categories	C-I (%)	C-II (%)	C-III (%)	C-IV (%)	C-I (%)	C-II (%)	C-III (%)	C-IV (%)	C-I (%)	C-II (%)	C-III (%)	C-IV (%)
Spider angioma *n* = 26	**10** **(38.4)**	10 (38.4)	6 (23)	—	**26** **(100)**	—	—	—	**—**	—	—	—
Facial telangiectesia *n* = 130	**8** **(6.15)**	40 (30.7)	50 (38.4)	32 (24.6)	**57** **(43.8)**	50 (38.4)	20 (18.5)	3 (2.3)	**125** **(97)**	2 (1.8)	2 (1.8)	1 (0.9)
Leg telangiectesia *n* = 99	**4** **(4.04)**	10 (10.1)	40 (40.4)	45 (45.4)	**21** **(21.2)**	59 (59.5)	12 (12.2)	7 (7.1)	**80** **(80.8)**	5 (5.5)	3 (3.3)	11 (11.1)

Efficacy categorized in groups: clear; Category(C)-I: marked improvement; C-II: partial response; C-III: poor response; C-IV: no change or worsening.
